# SJL Mice Infected with *Acanthamoeba castellanii* Develop Central Nervous System Autoimmunity through the Generation of Cross-Reactive T Cells for Myelin Antigens

**DOI:** 10.1371/journal.pone.0098506

**Published:** 2014-05-30

**Authors:** Chandirasegaran Massilamany, Francine Marciano-Cabral, Bruno da Rocha-Azevedo, Melissa Jamerson, Arunakumar Gangaplara, David Steffen, Rana Zabad, Zsolt Illes, Raymond A. Sobel, Jay Reddy

**Affiliations:** 1 School of Veterinary Medicine and Biomedical Sciences, University of Nebraska-Lincoln, Lincoln, Nebraska, United States of America; 2 Virginia Commonwealth University School of Medicine, Richmond, Virginia, United States of America; 3 University of Texas Southwestern Medical Center, Dallas, Texas, United States of America; 4 University of Nebraska Medical Center, Omaha, Nebraska, United States of America; 5 University of Pecs, Pecs, Hungary; 6 University of Southern Denmark, Odense, Denmark; 7 Stanford University School of Medicine, Stanford, California and VA Health Care System, Palo Alto, California, United States of America; Baylor College of Medicine, United States of America

## Abstract

We recently reported that *Acanthamoeba castellanii* (ACA), an opportunistic pathogen of the central nervous system (CNS) possesses mimicry epitopes for proteolipid protein (PLP) 139–151 and myelin basic protein 89–101, and that the epitopes induce experimental autoimmune encephalomyelitis (EAE) in SJL mice reminiscent of the diseases induced with their corresponding cognate peptides. We now demonstrate that mice infected with ACA also show the generation of cross-reactive T cells, predominantly for PLP 139–151, as evaluated by T cell proliferation and IA^s^/dextramer staining. We verified that PLP 139–151-sensitized lymphocytes generated in infected mice contained a high proportion of T helper 1 cytokine-producing cells, and they can transfer disease to naïve animals. Likewise, the animals first primed with suboptimal dose of PLP 139–151 and later infected with ACA, developed EAE, suggesting that ACA infection can trigger CNS autoimmunity in the presence of preexisting repertoire of autoreactive T cells. Taken together, the data provide novel insights into the pathogenesis of *Acanthamoeba* infections, and the potential role of infectious agents with mimicry epitopes to self-antigens in the pathogenesis of CNS diseases such as multiple sclerosis.

## Introduction

Multiple sclerosis (MS) is a chronic demyelinating inflammatory disease in which mononuclear cells (MNC) infiltrate the central nervous system (CNS) leading to the loss of oligodendrocytes and axonal degeneration [1]. There are no known etiological agents identified as triggers of MS nor is there permanent cure. It is widely believed that MS pathogenesis involves generation of autoimmune responses to myelin antigens requiring the mediation of T cells and B cells, but the underlying mechanisms are not well-understood [1], [2]. Two factors have been implicated in the predisposition to MS: a) genetic susceptibility and b) environmental microbes. The latter proposal has been supported by the observations that exacerbations of MS attacks or temporal alterations in the disease course occur after viral or bacterial infections, such as, with Epstein Barr virus, Human Herpes virus-6 and *Clostridium perfringens* type B. These associations have been made based on detection of microbial genomic material, and antibodies in the brains or CSF of MS patients [3]–[6]. In addition, peripheral blood MNC from MS patient subjects can also react to viral- and myelin basic protein (MBP)-specific peptide fragments [7], [8], but the direct causal links between these virus infections and predisposition to MS have not been proved clinically in a large number of cases [9]–[12]. A theme that has also being emerged suggests that MS trigger may involve exposure to multiple microbes [1], and their disease-mediation may involve more than one mechanism, such as bystander activation, release of cryptic epitopes, molecular mimicry and epitope spreading [13]–[15].

In our efforts to identify the disease-inducing microbial mimics for CNS myelin antigens, we recently identified two novel epitopes from *Acanthamoeba castellanii* (ACA) [16]–[18]. These are derived respectively from rhodanese-related sulfur transferase (RST), and nicotinamide adenine dinucleotide dehydrogenase subunit 2 (NAD) of *Acanthamoeba*. The corresponding mimics, designated ACA 83–95 and NAD 108–120, bear identical residues, respectively, for proteolipid protein (PLP) 139–151 and MBP 89–101. We showed that ACA 83–95 and NAD 108–120 induce experimental autoimmune encephalomyelitis (EAE) reminiscent of the diseases induced respectively with their cognate peptides through the generation of cross-reactive T cells [16]–[18]. We report here that SJL mice infected with trophozoites of ACA show the generation of myelin-reactive T cells. Furthermore, lymphocytes obtained from ACA-infected mice contained high frequencies of T helper (Th) 1 cytokine-producing cells that have the ability to transfer disease to naïve recipients.

## Materials and Methods

### Ethics Statement

Three-to-four-week-old female SJL/J (H-2^s^) mice were obtained from the Jackson Laboratory (Bar Harbor, ME). The mice were maintained in accordance with the animal protocol guidelines of the University of Nebraska-Lincoln, Lincoln, NE. The study was conducted in accordance with National Institutes of Health guidelines for the use of experimental animals, and the protocols were specifically approved by the University of Nebraska-Lincoln Institutional Animal Care and Use Committee (permit number: A3459-01; protocol # 454 and 659).

### Propagation of ACA, Infection Procedure, and Clinical Assessment

Human brain-isolate of ACA (strain 50494; ATCC, Manassas, VA) was grown in peptone-yeast extract-glucose medium (PYG medium, ATCC) in 75 cm^2^ flask at 25°C for four days. After replacing the medium, the trophozoites were grown for three additional days, and the adhered trophozoites were then harvested by gentle tapping and pipetting and subcultured for two days. Trophozoites were collected as above, and after washing twice, the pellet was resuspended in 1×Page’s saline that was warmed to room temperature (RT), and viability was confirmed [19]. For infection studies, mice were anesthetized using isoflurane and the indicated number of trophozoites was administered in 20 µl volume by instilling 10 µl into each nostril [20]–[22]. The animals that received only Page’s saline served as controls.

### Histopathology

Animals were euthanized on indicated days, and brain and spinal cords were collected in 10% phosphate buffered formalin for histology [17], [23]. After fixation, brain and spinal cord sections were processed routinely for paraffin embedment and sections stained with hematoxylin and eosin (H and E). The slides were evaluated by a neuropathologist who was blinded to treatment. The sections were scored for lesion type, and severity, and counts of inflammatory foci were determined in parenchyma and leptomeninges [17]. Total scores were obtained by adding counts of inflammatory foci in both meninges and parenchyma. Inflammation was primarily classified as lymphocytic, suppurative, or mixed [17]. All histological evaluations were performed by board-certified pathologists.

### Immunophenotyping

Two groups of mice were infected with ACA (1×10^3^), and the animals showing neurological signs were sacrificed to harvest brains and spinal cords. The tissues were processed to obtain MNC by percoll density-gradient separation [24], and the cells were stained with cocktails of antibodies for various immune cell markers, specifically, T cells (CD3, CD4 and CD8) and non-T cells (B220, CD11b, CD11c, GR1 and CD49b) (all antibodies from eBioscience, San Diego, CA), and 7-aminoactinomycin-D (7-AAD; Invitrogen, Carlsbad, CA). After acquiring by flow cytometry (FC) (FACSCalibur, BD Biosciences, San Diego, CA), percentages of cells positive for each marker were determined in the live (7-AAD^−^) population using Flow Jo software (Tree Star, Ashland, OR).

### Peptides

Myelin and mimicry peptide pairs (PLP 139–151, HSLGKWLGHPDKF; ACA 83–95, YFLLKWLGHPNVS: 53.18% similarity; and MBP 89–101, VHFFKNIVTPRTP; NAD 108–120, VVFFKNIILIGFL: 46.15% similarity; identical residues are underlined between peptide pairs); and Theiler’s murine encephalomyelitis virus (TMEV) 70–86 (WTTSQEAFSHIRIPLP) were synthesized on 9-fluorenylmethyloxycarbonyl chemistry (Neopeptide, Cambridge, MA). All peptides were HPLC-purified (>90%), identity was confirmed by mass spectroscopy, and the peptides were dissolved in 1×PBS as described previously [16]–[18].

### Proliferation Assay

Seven groups of mice were infected with ACA (5×10^3^), and T cell proliferative responses were measured using cells obtained from a pool of spleens and the draining lymph nodes (LN: cervical, mandibular axillary, and inguinal), hereafter designated ‘lymphocytes’, from ACA-infected animals. Where indicated, CD4 or CD8 T cells were purified from lymphocytes harvested from infected mice using CD4 or CD8 T lymphocyte enrichment kits (BD Biosciences, San Jose, CA). Cells were stimulated with PLP 139–151, ACA 83–95, MBP 89–101, NAD 108–120 or TMEV 70–86 (control) at a cell density of 5×10^6^ cells/ml for two days in growth medium containing 1×RPMI medium supplemented with 10% fetal calf serum, 1 mM sodium pyruvate, 4 mM L-glutamine, 1×each of non-essential amino acids and vitamin mixture, and 100 U/ml penicillin-streptomycin (Lonza, Walkersville, MD). In experiments involving CD4 T cells or CD8 T cells, splenocytes harvested from naïve SJL mice were irradiated (3000 rads; Rad Source, Suwanee, GA); loaded with peptides, and used as antigen presenting cells (APCs). After pulsing with tritiated ^3^[H] thymidine (1 µCi/well; MP Biomedicals, Santa Ana, CA) for 16 hours, proliferative responses were measured as counts per minute (cpm) using the Wallac liquid scintillation counter (Perkin Elmer, Waltham, MA). In some experiments, infected mice were injected with lipopolysaccharide (LPS) on day 20 and day 31 postinfection (25, 10 µg/mouse respectively) and the animals were euthanized on day 64 postinfection to purify CD4 T cells for proliferation assay.

### Creation of IAs/Dextramers, and Enumeration of the Frequencies of Antigen-specific T cells by Flow Cytometry

To determine the frequency of antigen-specific T cells, IA^s^/PLP 139–151, ACA 83–95 and TMEV 70–86 dextramers were created as described [17]. Briefly, the soluble IA^s^ monomers expressed in baculovirus were biotinylated using biotin protein ligase at a concentration of 25 µg/10 nmol of substrate (Avidity, Denver, CO). To prepare dextramers, biotinylated IA^s^ monomers were coupled to activated dextran backbones (kindly provided by Immudex, Copenhagen, Denmark) at 1∶20 molar ratio in 1×Tris Hcl 0.05 M, pH 7.2 for 30 minutes at RT as described previously [24]. Lymphocytes obtained from nine groups of ACA-infected (1×10^3^) mice were stimulated with PLP 139–151 or ACA 83–95 (50 µg/ml) for two days. Viable cells harvested on day 8 were incubated with allophycocyanin-conjugated IA^s^ dextramers at RT for two hours [24]. After washing twice, cells were stained with anti-CD4 and 7-AAD and acquired by FC. The frequency of dextramer-positive cells was then enumerated in live (7-AAD^−^) CD4^+^ population.

### Intracellular Cytokine Analysis

We determined the frequencies of Th1 (interferon [IFN]- γ), Th2 (interleukin [IL]-4, and IL-10), and Th17 (IL-17A, IL-17F, and IL-22) cytokine-producing cells based on intracellular staining by FC [17], [25]. Briefly, lymphocytes harvested from five groups of ACA-infected (1×10^3^) animals were stimulated with peptides (20 µg/ml) for two days, and the cultures were maintained in IL-2 medium. Viable cells harvested on day 5 or lymphocytes obtained from naïve mice, were stimulated for five hours with phorbol 12-myristate 13-acetate (PMA) (20 ng/ml) and ionomycin (300 ng/ml) (Sigma-Aldrich, St. Louis, MO) in the presence of monensin 2 mM (Golgi stop, BD Pharmingen, San Diego, CA). After staining with anti-CD4 and 7-AAD, cells were fixed, permeabilized and stained with cytokine antibodies or isotype controls (eBioscience). The frequency of cytokine-secreting cells was then determined in the live (7-AAD^−^) CD4^+^ subset by FC [17].

### Adoptive Transfer Experiments

Three groups of mice were infected with ACA (1×10^3^) and after 15 days, the animals were sacrificed and cell suspensions were prepared from lymphoid tissues as above. We used two protocols to generate primary T cell cultures: (i) cells were stimulated with concanavalin-A (con-A, 1 µg/ml) for two days. After 48 hours, viable cells harvested by ficoll density-gradient centrifugation (100×10^6^/mouse) were administered i.p. into groups of naïve SJL mice; and (ii) cells were stimulated with PLP 139–151 (20 µg/ml) for two days, and the cells were maintained in growth medium containing IL-2 for three more days. On day 5 poststimulation, viable cells (50×10^6^/mouse) were administered i.p. into groups of naïve SJL mice. Pertussis toxin (PT; 100 ng; List Biological laboratories, Campbell, CA) was administered i.p. on days 0 and 2 posttransfer, and the animals were monitored for clinical disease. At termination, animals were euthanized, and CNS tissues were collected for histology [16]–[18].

### Induction of EAE by ACA Infection in Animals Primed with PLP 139–151

Three groups of SJL mice were primed with suboptimal doses of PLP 139–151 (2.5 or 10 µg/mouse) in complete Freund’s adjuvant (CFA). Seven days later, one group of mice from each group were infected with ACA (1×10^3^ trophozoites) by intranasal administration as described above, while the other, uninfected group, served as control. We also used two other control groups namely, saline (1× Page’s saline)- and CFA-recipients (uninfected and infected). The animals were monitored for clinical signs of EAE or encephalitis and scored until day 60, and at termination, brains and spinal cords were collected for histology. The scoring scale used to evaluate clinical signs of EAE was as described previously [17], [26]: 0 - healthy; 1 - limp tail or hind limb weakness but not both; 2 - limp tail and hind limb weakness; 3 - partial paralysis of hind limbs; 4 - complete paralysis of hind limbs; and 5 - moribund or dead.

### Detection of ACA Genomic DNA by PCR

Total DNA was extracted from the brains of mice that received Con-A- or PLP 139–151-stimulated cells, and the DNA was subjected for PCR analysis using ACA-specific primers (forward, 5′-GGCCCAGATCGTTTACCGTGAA-3′); reverse, 5′-TCTCACAAGCTGCTAG GGAGTCA-3′) to yield an ∼500 bp amplicon of the small subunit of 18S rDNA as previously described [27], [28]. The PCR products were resolved in 1% ethidium bromide-stained agarose gel electrophoresis.

### Statistics

Wilcoxon rank sum test was used to compare differences in the severity of clinical and histological EAE in animals infected with or without PLP 139–151 priming. Differences in T cell proliferation and frequencies of cytokine-producing cells were analyzed by student’s t. p≤0.05 values were considered significant.

## Results

### Establishment of ACA-induced CNS Disease in SJL Mice

To delineate autoimmune events in ACA infection, we used human-isolate of *Acanthamoeba,* which has been previously shown to induce granulomatous encephalitis in mice [20], [22]. In a dose-response study, we noted that high doses (1×10^5^ and above) resulted in more than 66% mortalities within 10 days postinfection, while the animals that received low doses (1×10^4^ and below) tolerated well and the mortalities were low (4/28, 14.3% with 1×10^3^; 2/12, 16.6% with 1×10^4^). Nonetheless, regardless of dose, most of the infected animals showed general weakness, loss of body weight, and respiratory distress during the initial acute attack within approximately seven days postinfection, followed by neurological signs. These include, circling, trunk ataxia or paresis of the hind limbs with or without hyperactivity, dog-sitting posture with laterally extended hind limbs, twirling of neck (head-tilt), and loss of balance. Some of these signs such as, circling, ataxia, paresis, and twirling of neck have also been described in EAE mice [29].

**Figure 1:**
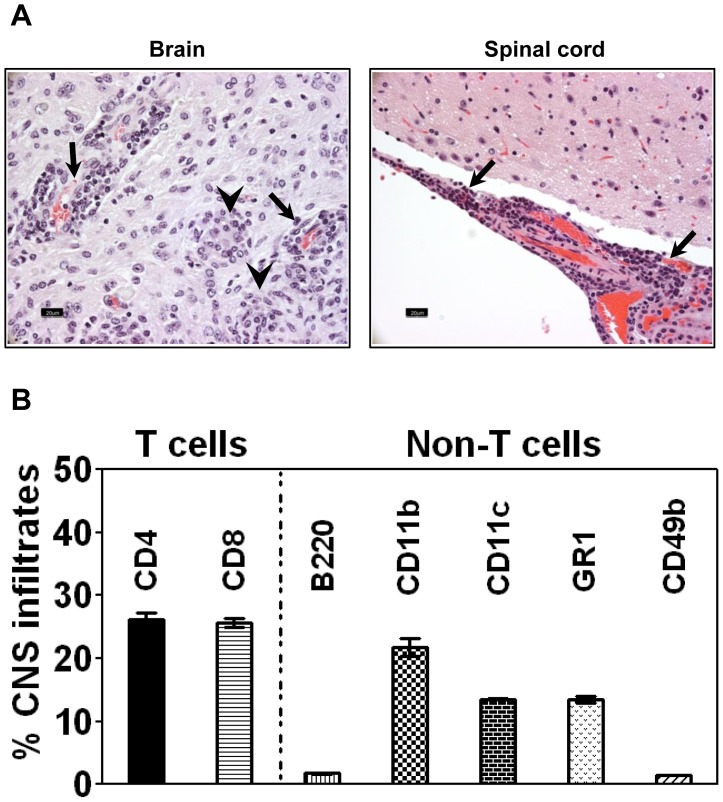
Evaluation of CNS inflammation in mice infected with ACA. (**A**) **Histological evaluation.** SJL mice were infected intranasally with ACA trophozoites (1×10^3^) under isoflurane anesthesia. Brains and spinal cords were harvested upon termination of the experiment, and the tissues were processed for CNS inflammation by H and E staining. Brain: Perivascular cuffing of lymphocytes (arrows) and granuloma-like lesions (arrowheads) composed of macrophages and lymphocytes. Spinal cord: Perivascular cuffing of lymphocytes in the leptomeninges (arrows). Original magnification, ×400 (bar = 20 µm). (**B**) **Immunophenotyping.** Groups of mice were infected with ACA as above, and animals showing neurological signs were killed on day 10 to harvest brains and spinal cords. The tissues were processed to obtain MNC by Percoll density-gradient separation, and the cells were stained with cocktails of antibodies for the indicated immune cell markers and 7-AAD. After acquiring the cells by flow cytometry (FC), percentages of various cell types were determined in the live (7-AAD^−^) cell population. Mean ± SEM values from two individual experiments involving two to three mice in each are shown.

We next evaluated CNS tissues for inflammatory changes. As shown in figure 1A, both meninges and parenchyma in the brains of majority of infected animals (60%) contained wide-spread perivascular lymphocytic cuffs. The animals affected with acute disease showed lesions mainly in the olfactory/frontal lobes; the infiltrates consisted of macrophages and lymphocytes, suggestive of granulomatous inflammation (Figure 1A). In contrast, only a few mice (10.7%) showed inflammation in the spinal cords. Likewise, staining with luxol fast blue did not reveal changes indicative of demyelination, although occasional myelin-disruption was evident (data not shown). Since most of the animals (more than 80%) that received a dose of up to 1×10^4^ survived the acute phase and showed histological evidence of inflammation, we chose the dose to be less than 1×10^4^ to determine autoreactive T cell responses during the course of ACA infection.

We then characterized the inflammatory cells using MNCs purified from the brains and spinal cords of ACA-infected mice. As shown in figure 1B, the infiltrates were comprised of both T cells and non-T cells, with T cells accounting for ∼51%, whereas non-T cells were represented by macrophages, dendritic cells, neutrophils, B cells and natural killer (NK) cells. Notably, the proportion of both CD4 and CD8 T cells were similar (26% vs. 25.6% respectively) suggesting that they may have a role in disease-mediation.

### SJL Mice Infected with ACA show the Generation of Myelin-reactive CD4^+^ T cells

**Figure 2:**
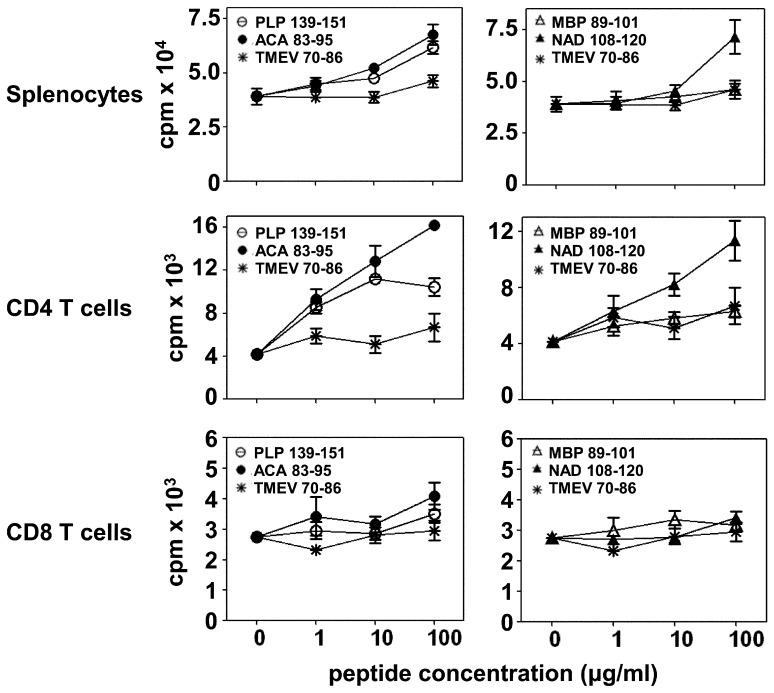
ACA infection leads to the generation of PLP 139–151-reactive T cells. Top panels: Groups of SJL mice were infected intranasally with ACA trophozoites (5×10^3^) and after 30 days, the mice were killed and spleens were harvested to prepare single cell suspensions. Cells were stimulated with PLP 139–151 or ACA 83–95 (top left panel), MBP 89–101 or NAD 108–120 (top right panel) for two days followed by pulsing with ^3^[H] thymidine, the incorporation of which was measured as cpm 16 hours later. TMEV 70–86, control. Mean ± SEM values from three individual experiments involving one mouse in each are shown. Middle and bottom panels: Lymphocytes were obtained from infected animals on day 10 post-infection and CD4 or CD8 T cells were then purified. Cells were stimulated with PLP 139–151 or ACA 83–95 (middle- and bottom left panels), MBP 89–101 or NAD 108–120 (middle- and bottom right panels) in the presence of irradiated APCs, and proliferative responses were measured as above. TMEV 70–86, control. Mean ± SEM values from one of the four individual experiments involving a group of five mice in each are shown.

Based on our previous data with ACA 83–95- and NAD 108–120-induced EAE, we hypothesized that ACA infection leads to the generation of cross-reactive cells for PLP 139–151 and MBP 89–101. To test this hypothesis, groups of mice were infected with ACA, and on day 30 postinfection, splenocytes were used to measure proliferative responses to myelin and ACA antigens. The respective peptide pairs were: PLP 139–151 vs. ACA 83–95 and MBP 89–101 vs. NAD 108–120. Expectedly, cells from ACA-infected mice responded in a dose dependent manner to both the amoebic peptides, ACA 83–95 (Figure 2, top left panel) and NAD 108–120 (Figure 2, top right panel) with a difference of ∼ two-fold higher than that was observed with the control peptide (TMEV 70–86). Similarly, cells also responded to the myelin peptide, PLP 139–151 (Figure 2, top left panel) with a 1.6-fold difference, but such a response was lacking for MBP 89–101 (Figure 2, top right panel). These findings suggest that RST and NAD, the respective protein sources for ACA 83–95 and NAD 108–120 are naturally processed, and the peptides are presented to the immune system. Of note, aged SJL mice have a tendency to carry PLP-reactive T cells in their naïve periphery [30]. Our studies involved young mice of 3 to 4 weeks old, in which, we did not observe the presence of endogenously-derived PLP-reactive T cells (figure S1). Thus, our data suggest that ACA infection has led to the appearance of PLP-reactive T cells.

Further, we verified whether the reactivity to myelin antigens lies in CD4 or CD8 T cell compartment, since the CNS tissues from ACA-infected mice had similar proportions of CD4 and CD8 T cells (Figure 1B), and infiltrations were predominantly seen in the brains, and not in the spinal cords similar to those reported in MBP-specific CD8 T cell-mediated EAE in C3HeB/FeJ mice [31], [32]. To verify this, CD4 or CD8 T cells were purified from ACA-infected mice, and their proliferative responses to myelin and amoebic antigens were tested as above. We noted that the antigen-reactivity was confined only to CD4 T cells (Figure 2, middle panels) but not CD8 T cells (Figure 2, bottom panels), although, in some experiments, low-grade responses were noted for MBP 89–101, but the results were not consistent (data not shown). Expectedly, CD4 T cells reacted to the amoebic peptides, ACA 83–95 (four-fold; Figure 2, middle left panel) and NAD 108–120 (three-fold; Figure 2, middle right panel), and a proportion of ACA-sensitized CD4 T cells also responded to PLP 139–151 (2.5-fold; Figure 2, middle left panel), but the response to MBP 89–101 was absent (Figure 2, middle right panel) leading us to determine their antigen-specificity.

### Cross-reactive T cell Responses Induced with ACA are Antigen-specific

**Figure 3:**
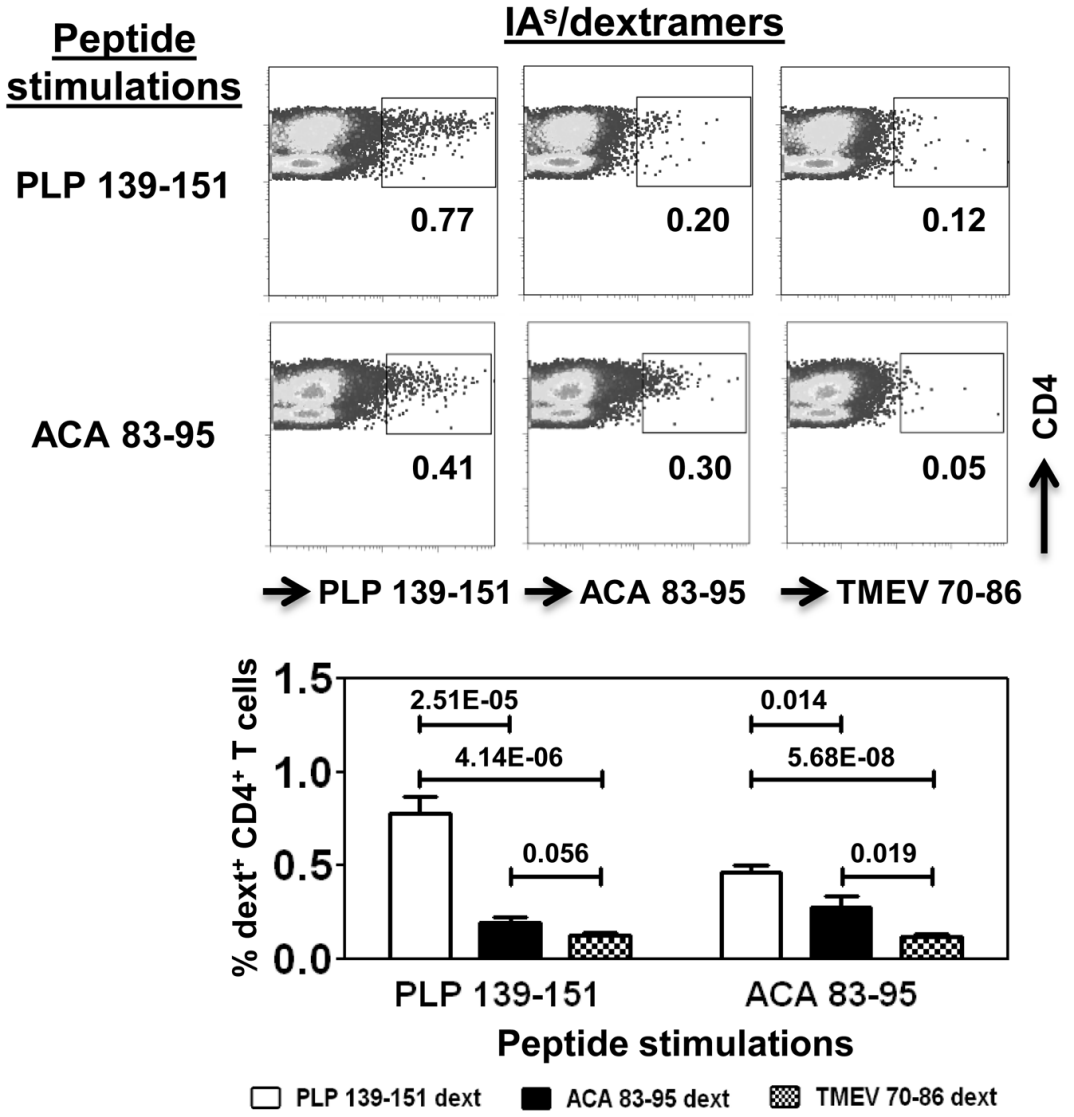
Cross-reactive T cell responses induced by ACA infection are antigen-specific. SJL mice were infected with ACA (1×10^3^) and after 21 days, the animals were killed; lymphocytes were prepared. Cells were stimulated with PLP 139–151 or ACA 83–95 for two days and the cultures were maintained in IL-2 medium. Cells were harvested on day 8 poststimulation with peptides, and stained with IA^s^/dextramers followed by anti-CD4 and 7-AAD. After acquiring the cells by FC, frequencies of dextramer-positive cells were determined in the live (7-AAD^−^) CD4 subset. Mean ± SEM values obtained from nine independent experiments involving three to five mice in each are shown.

To determine the antigen-specificity of cells that react with ACA and myelin antigens, we created major histocompatibility complex class II/IA^s^ dextramers for ACA 83–95 and PLP 139–151 as described previously [24]. Lymphocytes were harvested from ACA-infected animals, and the cells were stimulated with ACA 83–95 or PLP 139–151, in which, frequencies of antigen-specific cells were enumerated by FC. As depicted in figure 3, we noted that the mean frequency of ACA-specific cells in ACA 83–95-sensitized cultures was found to be 0.28±0.06% as opposed to 0.46±0.03% for PLP 139–151. Conversely, when the cells were stimulated with PLP 139–151, the mean frequencies of PLP-specific cells were estimated to be higher (0.78±0.09%) than that of ACA 83–95 (0.20±0.03%). The dextramer staining was specific, since the binding with control (TMEV 70–86) dextramers was negligible. The data suggest that ACA infection leads to the generation of T cells that cross-react with both amoebic and myelin antigens.

### Myelin-reactive T cells Generated in ACA-infected Mice Contain High Frequencies of Th1 Cytokine-producing Cells

**Figure 4:**
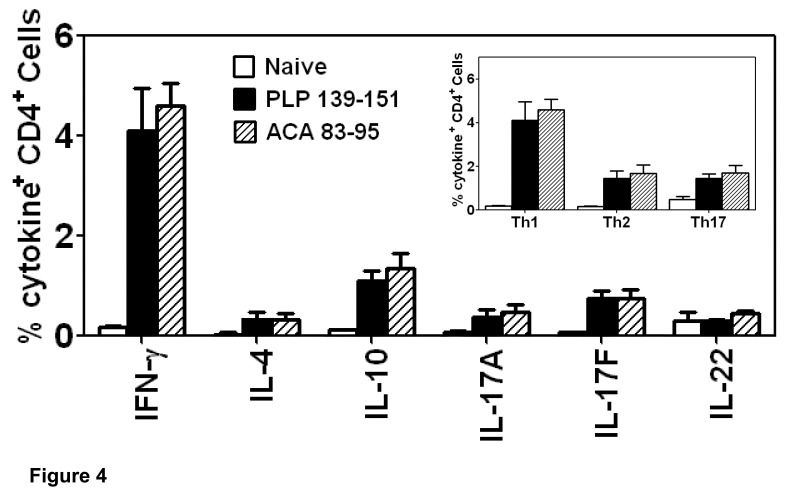
Antigen-sensitized lymphocytes from ACA*-*infected mice contain predominantly Th1 cytokine-producing cells. Groups of SJL mice were infected with ACA (1×10^3^). After 21 days, mice were killed, and lymphocytes were prepared. Cells were stimulated with PLP 139–151 or ACA 83–95 for two days and the cultures were maintained in IL-2 medium. Cells harvested on day 5 from the above cultures or those obtained from naïve mice were stimulated with PMA (20 ng/ml) and ionomycin (300 ng/ml) for ∼5 hours in the presence of 2 mM monensin followed by staining with anti-CD4 and 7-AAD. After fixation and permeabilization, cells were stained with cytokine antibodies and acquired by FC. Frequencies of cytokine-producing cells were then analyzed in the live (7-AAD^−^) CD4 subset. Inset: The combined frequencies of Th1 (IFN-γ), Th2 (IL-4+ IL-10) and Th17 (IL-17A+IL-17F+IL-22) cytokine-producing cells were calculated and compared between groups. Mean ± SEM values obtained from five independent experiments involving two mice in each are shown.

We used cytokines as readouts to evaluate the pathogenic potential of PLP 139–151-reactive T cells generated in ACA-infected mice. We assessed the frequencies of cytokine-producing CD4 T cells by intracellular staining [16]–[18]. These analyses included a panel of cytokines for Th1 (IFN-γ), Th2 (IL-4 and IL-10), and Th17 (IL-17A, IL-17F, and IL-22) cells. In both PLP 139–151- and ACA 83–95-sensitized cultures, cells capable of producing all the cytokines tested were present (Figure 4), but their frequencies differed in the order of Th1 (IFN-γ)>Th2 (IL-4+ IL-10) or Th17 (IL-17A+IL-17F+IL-22) cells (Figure 4; see inset). By deriving the ratios between the three Th cell subsets (Th1/Th2 and Th1/Th17), it was evident that the frequencies of IFN-γ-producing cells were higher by three-to-four-fold than Th2- or Th17-cytokine-producing cells (p<0.02). The cytokine responses are attributable to antigen-stimulations because lymphocytes obtained from naive SJL mice contained a negligible proportion of cytokine-producing cells (IFN-γ: 0.2%, Figure 4). The data suggest that ACA infection results in the generation of predominantly Th1-, but to a lesser extent, Th17 cytokine-producing cells, which are generally implicated in the development of organ-specific autoimmunity.

### Myelin Antigen-reactive Lymphocytes Generated in ACA-infected Mice Induce CNS Autoimmunity in Naïve Recipients

**Figure 5:**
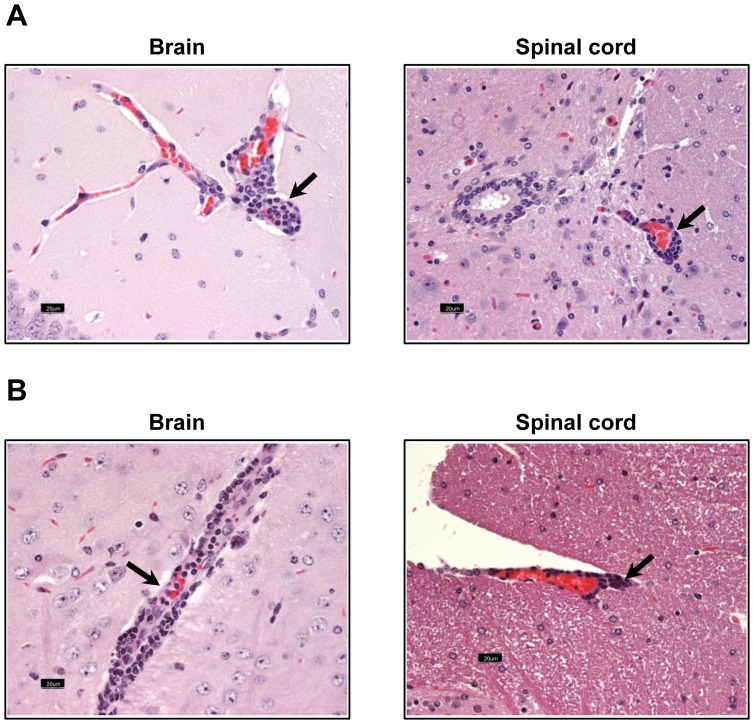
Lymphocytes from ACA-infected mice induce CNS inflammation in naïve mice. (**A**) **Con-A-stimulated cells.** Groups of SJL mice were infected with ACA (1×10^3^) and the animals were killed 15 days postinfection, and lymphocytes were prepared. Cells were stimulated with Con-A (1 µg/ml) for two days; viable cells were harvested and 100×10^6^ cells were injected i.p. into naïve mice. Brains and spinal cords were harvested on day 30 posttransfer, and the tissues were processed for CNS inflammation by H and E staining. Arrows indicate perivascular cuffing. Original magnification, ×400 (bar = 20 µm). (**B**) **PLP 139–151-stimulated cells.** Lymphocytes harvested as above were stimulated with PLP 139–151 (20 µg/ml) for two days and the cultures were maintained in IL-2 medium. Viable cells were harvested on day 5 poststimulation and 50×10^6^ cells were injected i.p. into naïve mice. Brains and spinal cords were harvested on day 45 posttransfer and processed for evaluation of CNS inflammation by H and E staining. Arrows indicate perivascular cuffing. Original magnification, ×400 (bar = 20 µm).

Based on the finding that antigen-sensitized lymphocytes generated in ACA-infected animals contained cells capable of producing Th1 and Th17 cytokines, we hypothesized that they can transfer disease into naïve recipients. To test this hypothesis, we isolated lymphocytes from ACA-infected mice and after expanding with con-A, cells were transferred into naïve SJL mice. Clinically, the recipients did not show clinical signs of EAE, but histologically, both brains and spinal cords showed inflammatory infiltrates. Although, immunophenotyping was not performed, inflammatory cells were deemed to be primarily lymphocytes and macrophages based upon morphology (Figure 5A, Table 1). Similar results were also noted in mice that received PLP 139–151-stimulated lymphocytes (Figure 5B, Table 1). These inflammatory changes were not due to the presence of ACA, if any, in the transfused cells, as the CNS tissues did not reveal the presence of ACA genomic DNA as evaluated by PCR analysis [27], [28] (figure S2). We thus, demonstrated that the myelin-reactive T cells generated in ACA-infected mice share the functional features of encephalitogenic T cells, and contribute to CNS disease.

**Table 1:** Histologic evaluation of EAE in mice that received lymphocytes from ACA-infected mice.

		No. of inflammatory foci†		
Treatment	Incidence† (%)	Meninges	Parenchyma	Total
Con-A	6/6 (100)	6.7±2.9	2.8±1.5	9.5±4.0
PLP 139–151	2/3 (66)	5.0±2.0	2.0±2.0	7.0±4.0

† represents mice that showed histological disease.

### ACA Infection can Alter Susceptibility to CNS Autoimmunity in Animals Primed with Suboptimal Doses of PLP 139–151

**Figure 6:**
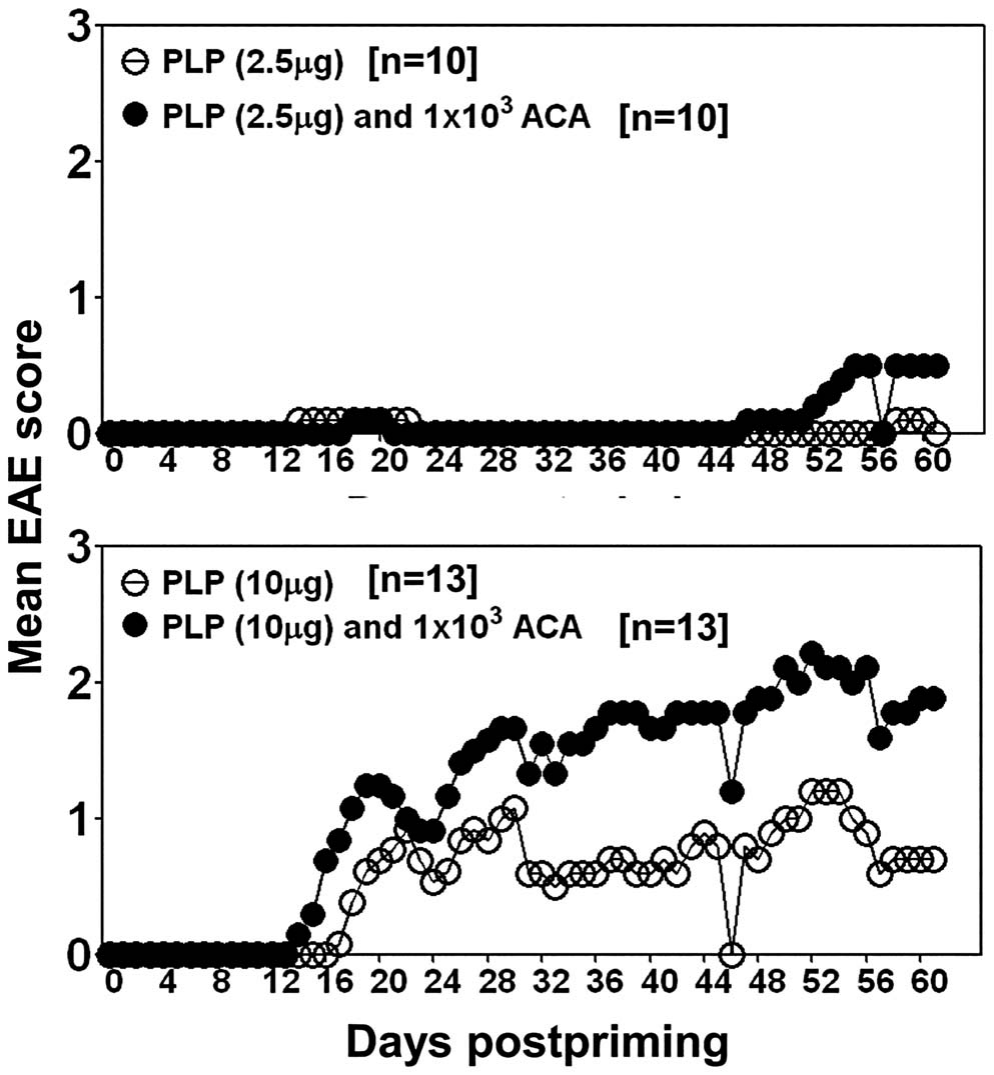
ACA infection alters susceptibility to EAE in mice primed with suboptimal doses of PLP 139–151. Groups of SJL mice were immunized with or without PLP 139–151 (2.5 or 10 µg/mouse) in CFA. On day 7 post-immunization, the animals were infected with ACA (1×10^3^) and the animals were monitored for clinical signs of EAE and scored. Data represent mean EAE scores for a group of mice pooled from two to three individual experiments.

To determine whether exposure to ACA can alter susceptibility to CNS autoimmunity in the presence of preexisting repertoire of autoreactive T cells, we immunized groups of mice with suboptimal doses of PLP 139–151 (2.5 or 10 µg/mouse) and the animals were monitored for EAE-signs after infecting them with ACA (1×10^3^/mouse). Figure 6 shows that animals that were primed with PLP 139–151, and later infected with ACA, developed clinical EAE with a greater severity as compared to the animals primed with PLP 139–151 alone, and the differences were clear in animals that received 10 µg peptide/CFA emulsion. We noted that the animals that received peptide/CFA emulsions alone did not develop EAE even up to day 16 postimmunization compared to those exposed to ACA (7.6%; 1/13 vs. 38.4%; 5/13) (Figure 6, lower panel). The daily median EAE scores for the group that received peptide/CFA alone remained less throughout the course of the study compared to the mice that were primed and infected with ACA (p = 0.037). The animals in control groups (saline or CFA) that were infected with or without ACA did not show EAE-signs (data not shown). Consistent with the clinical EAE, brains and spinal cords also showed a tendency for inflammatory foci to be increased in primed and infected mice compared to the mice that received peptide emulsions alone (Table 2). The data suggest that exposure to ACA can potentiate susceptibility to CNS autoimmunity in the presence of preexisting repertoire of autoreactive T cells.

**Table 2:** Histological evaluation of CNS tissues obtained at termination from ACA-infected mice primed with or without PLP 139–151.

Treatment groups			No. of inflammatory foci†		
PLP 139–151 priming (µg)	Infection	Incidence (%)	Meninges	Parenchyma	Total
2.5	−	4/5 (80)	16.5±2.9	3.8±1.3	20.3±4.1
2.5	+	4/4 (100)	21.3±4.1	11.8±5.8	33.0±9.6
10	−	8/8 (100)	62.3±29.7	71.1±30.3	133.5±50.8
10	+	7/8 (87.5)	85.0±27.6	92.4±34.9	176.0±49.6

† represents mice that showed histological disease.

### Administration of LPS into ACA-infected Mice Leads to the Generation of MBP 89–101-Reactive T cells

**Figure 7:**
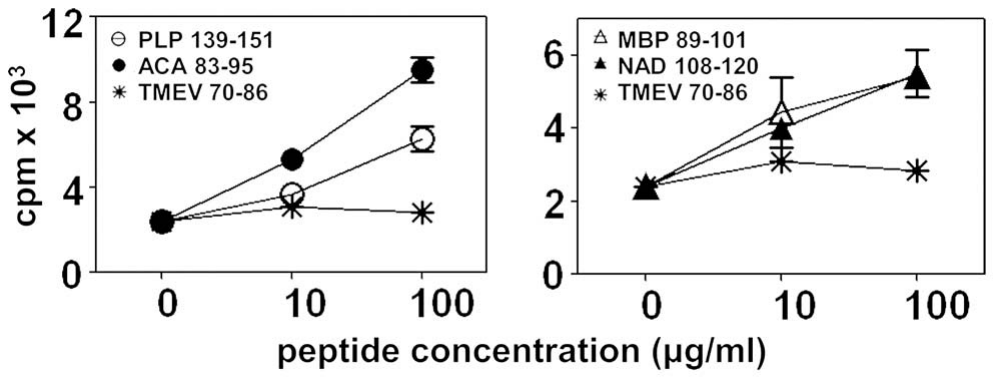
LPS-treatment of animals recovered from ACA infection leads to the generation of MBP 89–101-reactive T cells. Groups of SJL mice were infected intranasally with ACA trophozoites (7.5×10^3^) and on day 20 and day 31 postinfection, LPS was administered. Animals were killed on day 64 postinfection and lymphocytes were prepared to purify CD4 T cells. Cells were stimulated for two days with irradiated APCs loaded with peptides: PLP 139–151 or ACA 83–95 (left panel); and MBP 89–101 or NAD 108–120 (right panel). After pulsing with ^3^[H]-thymidine for 16 hours, proliferation was measured as cpm. TMEV 70–86 (control). Mean ± SEM values representing from one of the two experiments involving two mice in each are shown.

Microbial products such as LPS, bacterial DNA and unmethylated CpG’s have been shown to trigger autoimmunity by inducing the generation of effector Th1 cells under certain conditions [33]. We asked whether LPS-administration into animals that were recovered from ACA infection can promote the induction of EAE. ACA-infected mice were injected with LPS on day 21 and day 30 postinfection, and the animals were monitored for clinical signs of EAE. Contrary to our expectations, LPS-treatment did not lead to EAE during the observation period of ∼30 days. To verify whether this lack of disease induction is due to the absence of myelin-reactive T cells, we purified CD4 T cells from LPS-treated animals, and evaluated their responses to amoebic and myelin antigens using two peptide pairs (ACA 83–95/PLP 139–151 and NAD 108–120/MBP 89–101). We observed dose-dependent responses to both ACA 83–95 and PLP 139–151 as expected (Figure 7: left panel). Importantly, we also noted reactivity to MBP 89–101 in conjunction with the appearance of NAD 108–120-reactive T cells (Figure 7, right panel). Such reactivity to MBP 89–101 was absent in the infected animals that did not receive LPS (figure S3). Thus, our data support the notion that exposure to microbial infections irrelevant to CNS can lead to nonspecific activation of autoreactive T cells through the mechanism of bystander activation.

## Discussion

In this study, we provide four lines of evidence to demonstrate that ACA infection can predispose to CNS autoimmunity in SJL mice: a) mice infected with ACA show clinical and histological evidence of CNS inflammation by generating cross-reactive T cells for PLP139–151, b) ACA-sensitized lymphocytes have the ability to produce Th1 cytokines but to a low degree, Th17 cytokines that are known to be critical for induction of organ-specific autoimmune diseases [34], c) antigen-sensitized lymphocytes harvested from infected mice can transfer disease to naïve recipients, and d) ACA infection can alter susceptibility to EAE in animals primed with suboptimal doses of PLP 139–151.

EAE models generally have a limitation in that the disease is induced artificially with peptide-CFA emulsions. Hence, the immune events do not necessarily reflect those that can occur under natural conditions within the CNS. It has been proposed that exposure to environmental microbes can break self-tolerance leading to the recognition of myelin antigens as foreign. To test this hypothesis, it is critical to develop models that faithfully replicate the events of an infectious agent that primarily infects CNS, leading to the generation of autoimmunity secondarily, as might occur in ACA infection.

*Acanthamoeba* are free-living amoebae that are ubiquitous in the environment. Most healthy individuals carry *Acanthamoeba*-reactive antibodies, suggesting constant exposure to amoebae [21], [35], [36]. In spite of the high prevalence of the amoebae, the incidence of diseases caused by *Acanthamoeba* is very low. Non-opportunistically, Acanthamoebae can induce keratitis in healthy humans, but as an opportunistic pathogen, the amoebae can cause fatal encephalitis, especially, in immunocompromised individuals and treatments are often ineffective [21], [36]–[39]. We had recently reported that ACA contains mimicry epitopes for PLP 139–151 and MBP 89–101, and they induce EAE by generating corresponding cross-reactive T cells in SJL mice [16]–[18], [36]. These data prompted us to establish ACA infection in SJL mice to study the autoimmune events of *Acanthamoeba* infections.

Histological evaluation of CNS tissues obtained from ACA-infected mice revealed that brains but not spinal cords were consistently affected, and the inflammatory foci predominantly contained macrophages and lymphocytes. ACA is known to induce granulomatous encephalitis accompanied with hemorrhagic necrosis, but most studies used ‘death’ as the end-point to determine the pathogenicity [22], [40]–[43]. The dose used in our studies (1×10^3^) is ∼1000-fold less than that in other studies [22], [43]; and this amount of amoebae also led to fewer deaths but with no necrotic changes in the CNS. This suggests that the severity of the CNS pathology is directly proportional to the dose used to infect the mice. This notion is consistent with the finding that trophozoites were detected in less than 15% (4/28) of mice infected with 1×10^3^ ACA.

In EAE, there is a linear relationship between disease severity and permeability of the blood brain barrier (BBB), such that loss of BBB-integrity allows circulating immune cells and also soluble factors into CNS, and vice versa, for CNS products [44], [45]. *Acanthamoeba* can enter CNS by migrating through the olfactory neuroepithelium and/or blood [36]. Regardless of route however, the amoebae have to cross BBB paracellularly or transcellularly, where BBB-disruption is believed to be mediated by contact-dependent (via attachment) and contact-independent (via proteases) mechanisms [21], [36], [46]–[48]. Since, the inflammatory foci were commonly noted in the brains (especially, olfactory/frontal lobes), the tissue destruction most likely resulted from the damage directly caused by ACA.

On the contrary, the presence of inflammatory changes in the brains of infected mice, but in the absence of ACA trophozoites in most of them (24/28∶85.7%), indicates that the pathogenesis may involve the mediation of cellular events. Based on our previous data with ACA 83–95- and NAD 108–120 in EAE studies [16]–[18], [36], we reasoned that autoreactive T cells generated in response to ACA infection can contribute to CNS pathology in infected animals.

First, we verified that lymphocytes from infected animals reacted to both ACA 83–95 and NAD 108–120, suggesting that amoebic peptides are naturally processed and presented to the immune system. However, we noted that PLP-reactive- but not MBP-reactive T cells to be detected in the infected mice. Nonetheless, when MBP-reactive T cells were verified at various time points, day 5, 10, 15, 21, 30, and 82 postinfection, we noted low magnitude of responses to MBP 89–101, but the responses were not consistent. However, LPS-treatment of animals that recovered from ACA infection led to the appearance of MBP 89–101-reactive T cells but not EAE. We did not investigate whether this lack of disease is due to the inability of MBP-reactive T cells to infiltrate into the brains as intact BBB might have prevented their migration into the CNS. Reports indicate dual role for LPS in EAE pathogenesis in that the disease-severity could be suppressed or augmented depending on the stage of disease. For example, administration of LPS prior to EAE-induction led to the amelioration of EAE [49], [50]. Conversely, PLP 139–151-specific T cell receptor transgenic EAE-resistant B10.S mice, developed EAE spontaneously with LPS-treatment [51]. The disease-inducing abilities of LPS have been ascribed to its effects on APCs to upregulate costimulatory molecules, and secretion of soluble factors like IL-12, independent of T cell receptor-stimulation [33], [52].

We next determined whether myelin-reactive T cells that appear in ACA-infected animals are pathogenic, based on cytokine analysis and adoptive transfer experiments. We noted that lymphocytes from ACA-infected mice that are sensitized with PLP 139–151 were comprised of predominantly Th1- and to a lesser degree, Th17- and Th2 cytokine-producing cells. Although antigen-sensitized lymphocytes harvested from infected mice induced CNS inflammation in naïve recipients, clinical EAE was absent. Accumulated literature suggests that, while, both Th1 and Th17 cytokines can mediate CNS autoimmunity, Th17 cytokines appear to be indispensable, since IL-17- but not IFN- γ-deficient mice resist development of EAE [53], [54]. Additionally, sequential entry of Th cells may determine the outcome of EAE in that, Th1 cells migrate into the CNS prior to Th17 cells, which, then promote chemotaxis, inflammation and demyelination [55], [56]. Thus, the lack of typical EAE signs in animals that received antigen-stimulated cells may be due to insufficient production of Th17 cytokines. However, the observation that, ACA infection led to the induction of EAE in animals primed with suboptimal doses of PLP 139–151, point to a possibility that the pre-existing repertoire of autoreactive T cells as might occur in genetically susceptible individuals may expand in response to *Acanthamoeba* infections leading to CNS autoimmunity. Consistent with this notion, the clinical EAE was apparent in the animals that received 10 µg of peptide as compared to those that received 2.5 µg. This is likely because, *Acanthamoeba* carries a cross-reactive epitope for PLP 139–151, and as such, proportion of cross-reactive T cells are expected to be more in animals injected with 10 µg than 2.5 µg of peptide.

In summary, we demonstrate that ACA infection can lead to the generation of encephalitogenic cross-reactive T cells by antigenic mimicry, thus establishing a novel disease model to study CNS autoimmune diseases like MS. ACA is ubiquitously present in the environment worldwide, having been isolated from wide-range of sources, including soil, water and eye-wash stations and as contaminants; and the importance of ACA is increasingly being recognized in the acquisition of nosocomial infections [21], [36]. Although, no associations have been reported between ACA infection and MS prevalence, MS has been considered in the differential diagnosis of *Acanthamoeba* encephalitis [57]. Furthermore, serologic evidence suggests that, more than 50% of healthy humans can carry *Acanthamoeba*-reactive antibodies and seropositivity occurred in the order of Caucasians followed by Hispanics and African Americans [19], [35], [47], [58], [59]. In addition, T cells from healthy individuals can react to *Acanthamoeba* antigens, and antigen-specific T cell clones capable of secreting IFN-γ have been derived [60]. Likewise, peripheral blood MNC from rheumatoid arthritis patients show proliferative responses to *Acanthamoeba*, and amoebic encephalitis can occur in patients with systemic lupus erythematosus; the significance of these findings is not known [35], [58], [59], [61]. Our data may present a compelling rationale to investigate the role of ACA in MS-predisposition, which we are currently investigating. Our preliminary results indicate that cerebrospinal fluid samples obtained from MS patients, but not from those with other neurological disorders, show evidence of ACA-genomic material as evaluated by PCR (unpublished observations). Further as we reported previously [16], by deriving homology model for NAD 104–118 complexed with human leukocyte antigen-DR2 molecule, NAD 104–118 has the potential to induce cross-reactive T cells for human MBP 85–99, which is recognized as one of the major immunodominant epitopes in MS patients [62]. Demonstrating that CNS pathogens of humans like ACA have a role in MS predisposition may create opportunities to target the inciting agents of MS therapeutically.

## Supporting Information

Figure S1 **Analysis of endogenously derived PLP-reactive T cells in naïve SJL mice. (A) Proliferative response.** Splenocytes were prepared from naïve SJL mice aged 3 to 4 weeks, and the cells were stimulated with PLP 139–151 and TMEV 70–86 (control) for two days. After pulsing with ^3^[H]-thymidine for 16 hours, proliferation was measured as cpm. Mean ± SEM values from three individual experiments involving two mice in each are shown. **(B) Dextramer staining.** CD3^+^ T cells enriched from naïve mice were stained with PLP 139–151 or TMEV 70–86 (control) dextramers, anti-CD4 and 7-AAD. After acquiring the cells by FC, frequencies of dextramer-positive cells were determined in the live (7-AAD^−^) CD4 subset. Top panels, representative FC plots. Bottom panel, mean ± SEM values from six experiments each involving one to three mice are shown.
(TIF)

Figure S2 **PCR analysis of ACA genome in the brains of naïve recipients of cells derived from mice infected with** ***A. castellanii*** **.** Total DNA was extracted from the brains of mice that received Con-A- or PLP 139–151-stimulated cells, generated from animals infected with *A. castellanii.* After subjecting the DNA for PCR analysis using *A. castellanii*-specific primers, the PCR products were resolved in 1% agarose gel electrophoresis and stained with ethidium bromide (n = 9).
(TIF)

Figure S3 **Analysis of autoreactive T cells in mice chronically infected with** ***A. castellanii*** **.** SJL mice infected with ACA trophozoites (20×10^3^) were euthanized 12 weeks postinfection, and CD4 T cells were enriched. After stimulating the cells with the indicated peptides in the presence of irradiated APCs for two days, cells were pulsed with ^3^[H]-thymidine for 16 hours, and proliferation was then measured as cpm. TMEV 70–86 (control). Mean ± SEM values from three individual experiments are shown.
(TIF)
