# Supplementary figures and images for: SJL Mice Infected with Acanthamoeba castellanii Develop Central Nervous System Autoimmunity through the Generation of Cross-Reactive T Cells for Myelin Antigens
Source: PLoS One. 2014 May 30;9(5):e98506. doi: 10.1371/journal.pone.0098506 (PMC4039519; doi:10.1371/journal.pone.0098506)

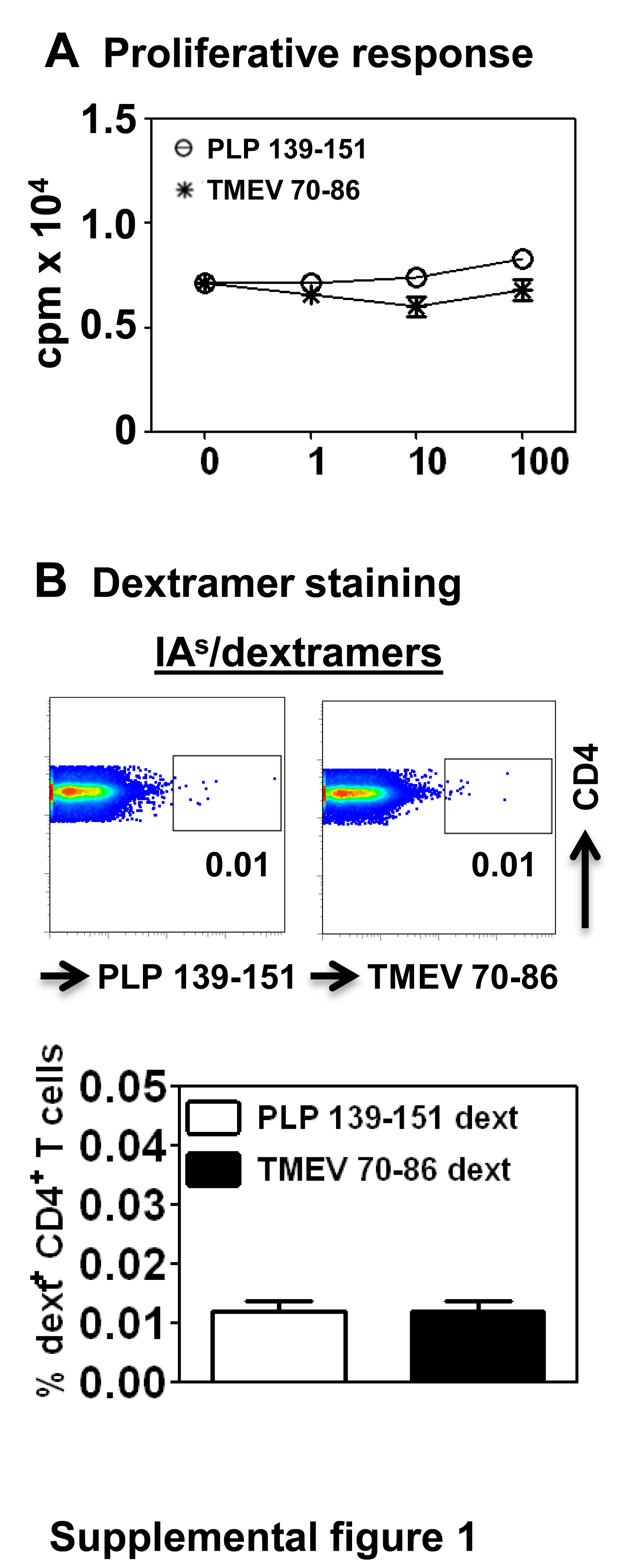

Supplement: Figure S1 — Analysis of endogenously derived PLP-reactive T cells in naïve SJL mice. (A) Proliferative response. Splenocytes were prepared from naïve SJL mice aged 3 to 4 weeks, and the cells were stimulated with PLP 139–151 and TMEV 70–86 (control) for two days. After pulsing with 3[H]-thymidine for 16 hours, proliferation was measured as cpm. Mean ± SEM values from three individual experiments involving two mice in each are shown. (B) Dextramer staining. CD3+ T cells enriched from naïve mice were stained with PLP 139–151 or TMEV 70–86 (control) dextramers, anti-CD4 and 7-AAD. After acquiring the cells by FC, frequencies of dextramer-positive cells were determined in the live (7-AAD−) CD4 subset. Top panels, representative FC plots. Bottom panel, mean ± SEM values from six experiments each involving one to three mice are shown. (TIF) [file pone.0098506.s001.tif]

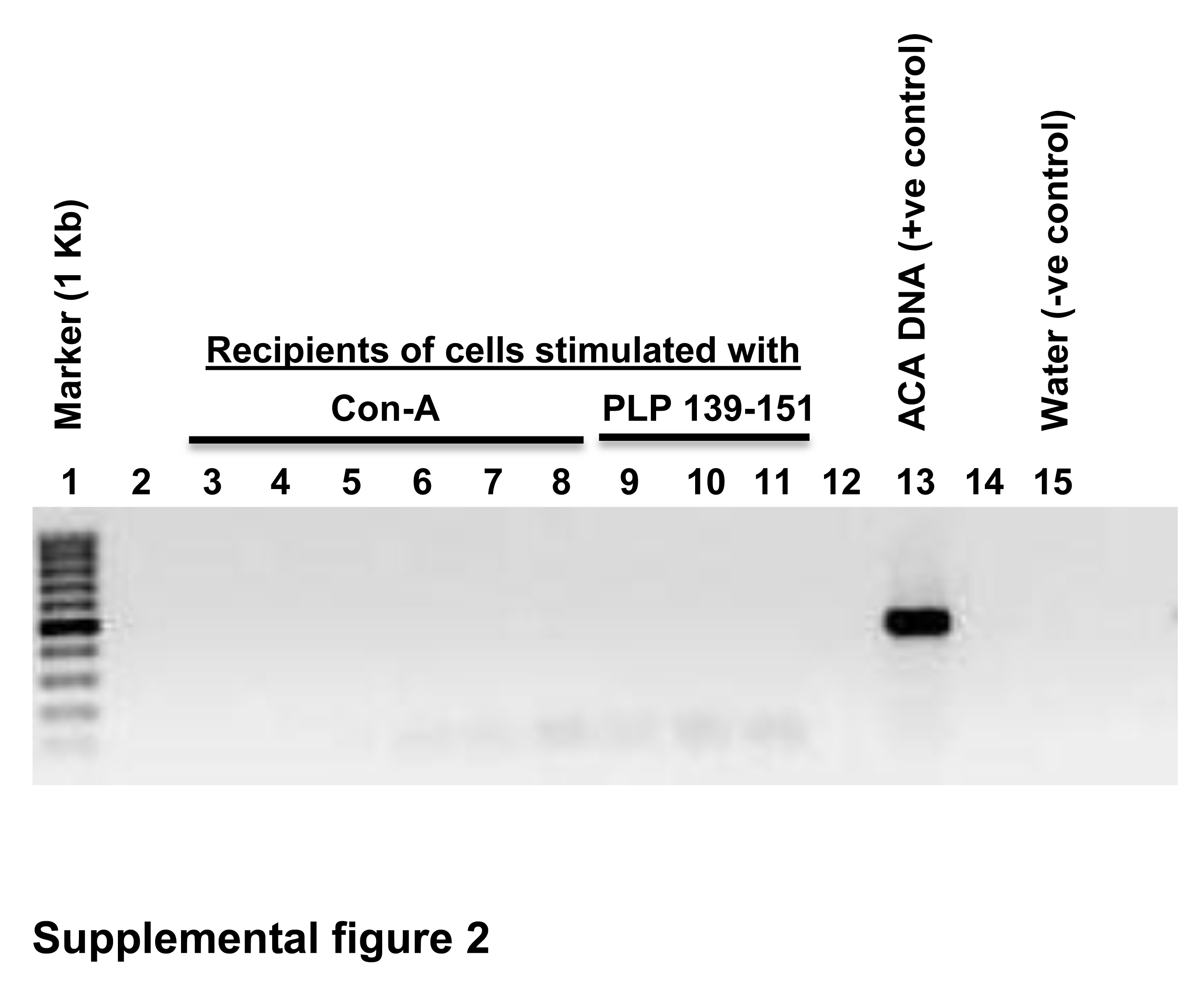

Supplement: Figure S2 — PCR analysis of ACA genome in the brains of naïve recipients of cells derived from mice infected with A. castellanii . Total DNA was extracted from the brains of mice that received Con-A- or PLP 139–151-stimulated cells, generated from animals infected with A. castellanii. After subjecting the DNA for PCR analysis using A. castellanii-specific primers, the PCR products were resolved in 1% agarose gel electrophoresis and stained with ethidium bromide (n = 9). (TIF) [file pone.0098506.s002.tif]
